# Graphene-based optical waveguide tactile sensor for dynamic response

**DOI:** 10.1038/s41598-018-34613-2

**Published:** 2018-10-31

**Authors:** Jin Tae Kim, Hongkyw Choi, EunJin Shin, Suntak Park, In Gyoo Kim

**Affiliations:** 1 Electronics and Telecommunications Research Institute (ETRI), 218 Gajeongro, Yuseong, Daejeon 34129 Republic of Korea; 2 ICT, ETRI School, University of Science and Technology, 218 Gajeongro, Yuseong, Daejeon 34129 Republic of Korea

## Abstract

Optical tactile sensors based on a directional coupler have been widely investigated because of their many advantages. However, one important requirement limits their wide application: the refractive index of the upper superstrate must be equal to or larger than that of the optical waveguide core. To overcome this disadvantage, an optical waveguide tactile sensor using graphene is proposed and its operational feasibility was validated experimentally. The pressure-dependent lateral deformation of the low-index prism-like microstructure on an elastomer superstrate has a key role in optically measuring the mechanical pressure. By mechanically varying the lateral deformation area, the waveguide core-graphene-polydimethylsiloxane (PDMS) interface area was adjusted and the amount of light absorption by graphene became tunable, even when the refractive index of the superstrate was lower than that of the waveguide core. The dynamic response of the sensor was accurately matched to the repeated pressing and release time of the pressure, and exhibited a real-time response to multi-stepped mechanical pressing and releasing using a piezoelectric motor. The proposed graphene-based optical tactile sensor is foundational to the use of a wide range of materials for overcoming the shortcoming of a directional coupler-based optical tactile sensor.

## Introduction

Recently, extensive research efforts have been concentrated on the application of tactile sensors to various fields such as soft robotics^1^, smart medical diagnosis^2^, real-time healthcare monitoring^3^, smart windows^4^, displays^5^, security systems^6^, mobile phones^7^, and electronic skins (e-skins)^8^. In comparison with electrical sensors based on the mechanisms of piezo-resistivity^9^, piezoelectricity^10^, and capacitance^11^ optical sensors have attracted a great amount of attention because of their numerous advantages, such as high immunity to electromagnetic interference, higher sensitivity, and longer lifetimes.

Optical tactile sensor array technologies have been investigated on the basis of the directional coupler principle^12,13^. By mechanically touching or releasing a superstrate on an air-cladding optical waveguide, the intensity of the output optical power can be adjusted dynamically. These tactile sensors provide an accurate optical response to the physical pressure exerted on the system. However, in this method, a restriction exists with regard to changing the traveling path of light or suppressing the propagation of the lightwave, whereby the refractive index of the upper substrate must be equal to or larger than the refractive index of the optical waveguide core. This constraint results in the drawback of limiting the optical materials that can be used in the design and manufacturing of optical tactile sensors to specific materials.

In this study, to overcome the disadvantages of the directional coupler-based optical tactile sensor, a novel optical tactile sensor module using a graphene material and elastomeric superstrate was designed and validated experimentally. The pressure-dependent lateral deformation of the prism-like microstructure has an important role in measuring the physical pressure by means of an optical method. The application of the vertical mechanical force to an elastomer superstrate, namely, polydimethylsiloxane (PDMS), which has a long prism-like microstructure, was placed on a graphene film integrated into an optical waveguide, and deformed the prism-like microstructure laterally. Then, the graphene-elastomer interface became wider and the guiding light in the waveguide core was subjected to high attenuation because of the graphene. This resulted in reduced optical power attenuation, even when the refractive index of the superstrate was lower than that of the waveguide core. To confirm the dynamic optical response to the various mechanical forces, the temporal behaviors of the mechanical-to-optical transducer were rigorously exploited. We found that the theoretical predictions and the experimental results of the device were in good agreement.

## Results and Discussion

**Figure 1:**
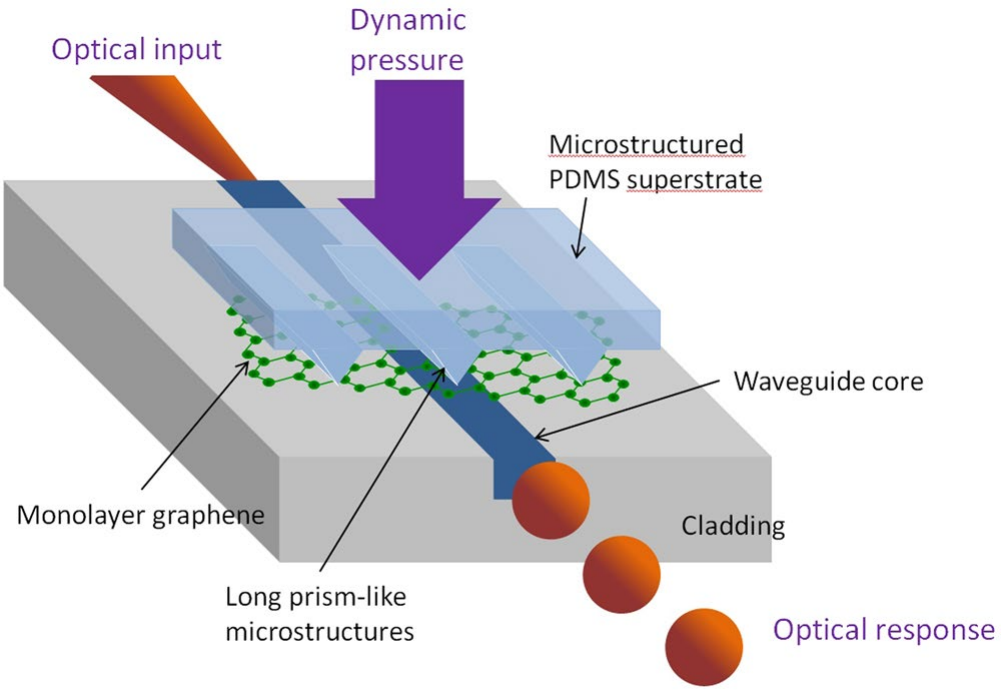
Bird’s eye view of the graphene-based optical waveguide tactile sensor. An elastomeric polydimethylsiloxane (PDMS) superstrate with straight prism-like microstructures was placed on the graphene-integrated optical waveguide platform. By mechanically varying the lateral deformation area, the waveguide core-graphene-PDMS interface area was adjusted and the amount of light that was absorbed by the graphene could be adjusted, even when the refractive index of the superstrate was lower than that of the waveguide core. The microstructures were fabricated by using a Si micro-channel platform formed by an anisotropic Si wafer wet etching process.

**Figure 2:**
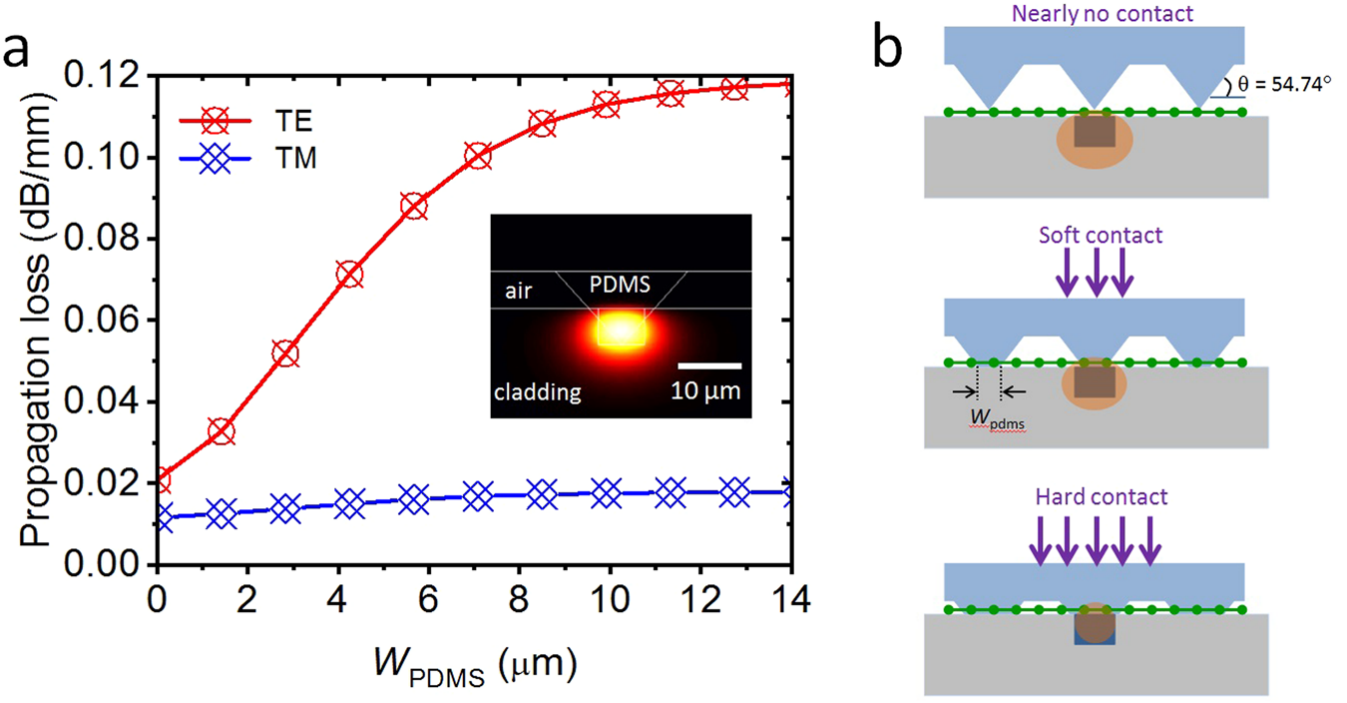
(**a**) Calculated propagation loss of the TE and TM modes as a function of area *W*_pdms_ and the covering width by the deformed polydimethylsiloxane (PDMS) microstructure. The inset shows a cross-sectional view of the designed device and the field distribution of the calculated guided mode, which indicates the fundamental mode propagation of the guided modes. (**b**) Working principle of a force sensor that detects the input force applied to the microstructured PDMS superstrate.

The configuration of the graphene-based optical waveguide tactile sensor is shown in Fig. 1a. The designed optical device consisted of an optical waveguide platform, graphene integrated on the platform, and elastomeric PDMS superstrate with straight prism-like microstructures. One edge corner of the prism-like structure was positioned at the center of the graphene-waveguide core interface. The other microstructures were placed on the side of the core to serve as elastomeric spacers. The prism-like microstructures were fabricated by casting a solution of PDMS onto a preformed Si wafer mold consisting of arrays of the inverse of the features to be replicated. The slope (blaze) angle of 54.7° in the PDMS microstructure replica (top of Fig. 2b) was produced by using the natural characteristics of anisotropic etching of a Si substrate whose (100) crystal angle plane was exposed to the wet etchant potassium hydroxide (KOH)^14^. As previously reported, numerous pressure sensors rely on this prism-like PMDS microstructure replica and pyramid-like microstructure array^15^.

The working principle of the device was based on the different graphene-light interactions, depending on the presence or absence of the superstrate (upper-cladding) upon the graphene-integrated optical waveguide platform^16^. Because of its light absorption characteristics^17^, the light wave that propagated along the waveguide core was subjected to power attenuation if the graphene film was present on the waveguide platform. The optical attenuation strength was further enhanced when the upper part of the graphene film was covered with a superstrate such as UV-curable polymer resin because of the increase in the optical intensity of the guided mode at the graphene-superstrate interface^16^. In ref.^16^, the averaged insertion loss of the transverse-electric (TE) mode changes from 10.9 dB to 50 dB when the graphene on the waveguide core is covered with a UV-curable polymer resin upper-cladding. The insertion loss of the transverse-magnetic (TM) modes also increases slightly. The alteration of the optical insertion loss can be achievable by the presence of the ion-gel on the single-layer graphene^18^. If mechanical attachment and detachment of the superstrate (upper-cladding) and graphene was repeatedly possible and if the relation between the optical output power and the change in the vertical mechanical force was qualified, an optical pressure sensor using graphene can be realized. However, the UV-curable polymer resin or ion-gel superstrate is not a suitable material for this purpose because it is permanently attached to the graphene film. A flexible and non-sticky optical superstrate is highly demanded.

To satisfy the requirement, we used a microstructured elastomer superstrate. In the proposed photonic device, the lateral deformation of the prism-like structure by a vertical mechanical force resulted in the desired effect. i.e., the repeated covering or exposure of the graphene film, and the detection of mechanical force variations using an optical approach. Thus, the shortcoming which arises from the usage of the immobile superstrate could be effectively addressed. As the vertical mechanical force is increased, the lateral deformation area also increases. The magnitude of the light-graphene interaction is therefore enhanced, and consequently, the optical output power at the waveguide end-facet decreases with the increase of the pressing pressure. The device operates even if the refractive index of the PDMS is lower than that of the waveguide core because the principle of operation is based on the variation of the light-graphene interaction via the adjustment of the graphene-covering area.

The release of the applied vertical mechanical force led to the restoration of the elastomeric PDMS microstructure, owing to its spring-like compressibility. Then, the attenuated output optical power returned to the initial state. If we provided the PDMS superstrate with periodical or discrete pressure, the amplitude of the output optical power could be temporally modulated or continuously attenuated, which indicates that the optical device can serve as an optical modulator or optical attenuator.

To theoretically confirm the working principle, we first calculated the optical characteristics of the guided mode as a function of the cover area (*W*_pdms_) of the prism-like microstructure, which deformed at the graphene-waveguide core interface (center of Fig. 2b). Theoretical investigations were conducted at the wavelength of 1.55 μm by using the PhotonDesign finite element method simulation software. The width and height of the waveguide core was 7 μm and 7 μm, respectively. The refractive indices of the core and cladding were 1.455 and 1.45, respectively. The refractive index of the PDMS was 1.396, which is less than that of the waveguide core. The graphene’s refractive index was calculated by using a complex form of the Kubo formula in^19,20^, and by taking into account the fact of graphene being an anisotropic material.

Figure 2a shows the calculated propagation loss of the TE and TM modes as a function of area *W*_pdms_ and the covering width by the deformed PDMS microstructure. The inset shows a cross-sectional view of the designed device and the field distribution of the calculated guided mode, which indicates the fundamental mode propagation of the guided modes. Without pressure (top in Fig. 2b, *W*_pdms_ = 0), the contact of the very narrow part of the edge of the prism-like structure with graphene allowed for the upper part of the graphene to be exposed to air. The optical loss caused by graphene was comparatively small. Because of the polarization-dependent nature of graphene, the propagation loss of the TE mode was higher than that of the TM mode^17,18^.

The propagation loss of the TE mode began to increase as (*W*_pdms_) increased, which means that vertical mechanical force was given. According to our assumption, the field amplitude and intensity of the guided mode at the waveguide core-graphene-PDMS interface increased. Then, the light was subjected to stronger power attenuation by the graphene, which resulted in the increased propagation loss of the guided mode. The optical power at the waveguide’s output facet was relatively reduced. The further application of the vertical mechanical force led to a wider contact area of the deformable PDMS material with graphene film (it induced the hard contact shown at the bottom of Fig. 2b). The width of the deformed microstructure was larger than that of the waveguide core, and extremely in excess of the width of the optical waveguide core. Although a further increase of the vertical mechanical pressure could have occurred, a certain maximum value was reached. As *W*_pdms_ increased, the field amplitude of the guided mode reaching into the PDMS microstructure also increased. However, the increase of the field amplitude was limited because of the fixed index contrast between the core, cladding, and PDMS. The optical response of the TE mode to the variation of *W*_pdms_ (and hence to the pressure change) was more sensitive than that of the TM mode, owing to the polarization-dependent nature of the graphene’s light absorption coefficient^17,18^.

**Figure 3:**
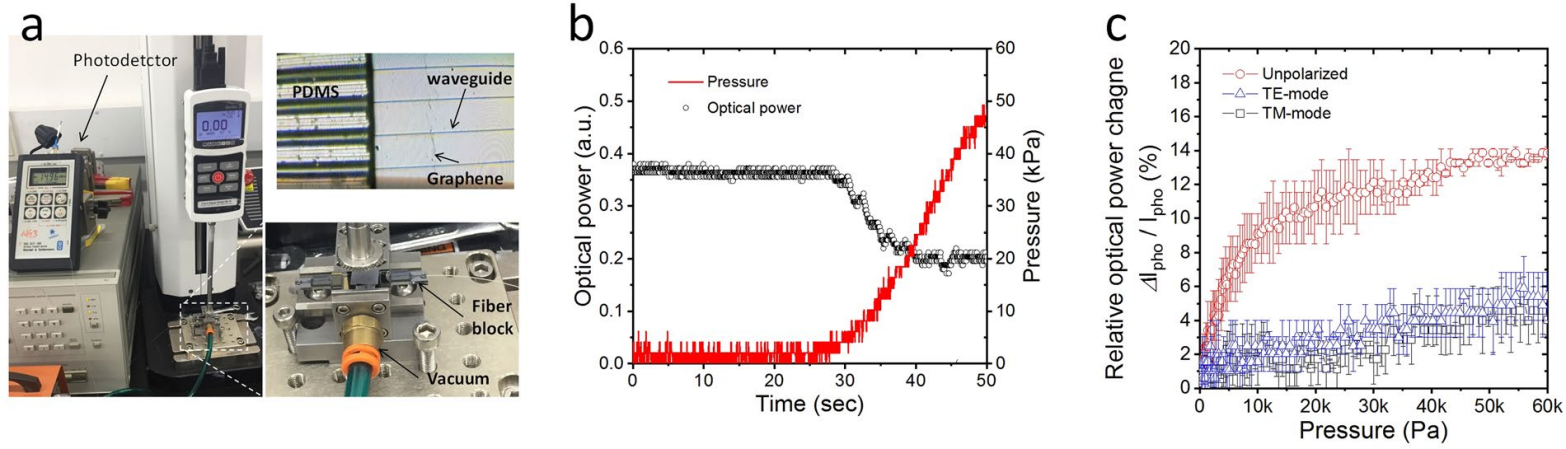
(**a**) View of the measurement setup for the proposed graphene-based optical waveguide tactile sensor. The optical tactile sensor is placed in a stable position on the vacuumed sample stage (right bottom inset) and the optical output power is measured using a photodetector. Microscope image of the fabricated optical tactile sensor (right top inset) indicates that one edge of the prism-like structure was accurately aligned on the waveguide core. (**b**) Light intensity measured at the output facet of the optical device and the amount of vertical pressure as a function of time. The optical output power decreases with the increase of pressure and is saturated at pressure of 40 kPa. (**c**) The relative optical power change as a function of pressure. As the mechanical pressure increased, the optical output power decreased (relative optical power change increased). The relative optical power change of the TE mode was greater than that of the TM mode since the TE mode light was more absorbed by the single layer graphene owing to the polarization-dependent nature of the graphene’s optical absorption coefficient.

Figure 3a shows a view of the measurement setup using a commercially available force gauge instrument (Mark-10, ESM 303 tensile compression force tester and M5–10 force gauge). The optical tactile sensor is placed in a stable position on the vacuumed sample stage (right bottom inset) and the optical output power is measured using a photodetector. A mechanical vertical force was applied by a vertical mechanical movement of the long bar attached the force gauge, whose upward and downward movements of the bar that exerted a certain pressure on the PDMS superstrate. A plat plate was attached at the end of the long bar, and a Si piece was placed on the PDMS superstrate for uniform pressure. The input and output fibers were tightly bonded to the waveguide sample to prevent misalignment between the sample and fiber during the application of the vertical force, as shown in the right bottom inset of Fig. 3a. The microscope image of the fabricated optical tactile sensor shown in the right top inset in Fig. 3a indicates that the graphene film was formed on the waveguide platform, and one edge of the prism-like structure was accurately aligned on the waveguide core. The background signal from the polymer substrate made it difficult to obtain the Raman shift of graphene on the waveguide platform. We measured the Raman shift (including the excitation wavelength at 514 nm) of the same graphene film on a SiO_2_/Si substrate. Based on the comparison of the 2D and G peak intensities measured at 2,700 and 1,580 cm^−1^, respectively, we concluded that an approximately monolayer graphene (the 2D peak intensity was larger than that of the G peak) with some defects was successfully transferred onto the polymer waveguide platform. A detailed description of the characteristics of the graphene film can be found in our previous work^18^.

Figure 3b shows light intensity measured at the output facet of the optical device and the amount of vertical pressure as a function of time. When pressure is not applied, the variation of the output optical power is subtle. Slight light intensity variation is attributed to negligible instability of the laser diode (LD) light source, which was carefully controlled by the combination of the laser diode and thermoelectric cooler (TEC) controller (Newport). The optical output power begins to slowly decrease when pressure is applied to the sample. As we predicted, the optical output power decreases with the increase of pressure. The optical power attenuation begins to be saturated at 30 kPa pressure and more power decrease is not detected under pressure of larger than 40 kPa.

Figure 3c shows the relative optical power change (*Δ*I_pho_/I_pho_) of an optical device that varies when pressure is applied to the upper substrate as the difference (*Δ*I_pho_) between the optical power without pressure (I_pho_) and the optical power under pressure (I_pho,force_). The change in the optical power (*Δ*I_pho_ = I_pho_ − I_pho,force_) corresponds to the strength of the vertical pressing force. As the mechanical pressure increased, the optical output power decreased (the relative optical power (*Δ*I_pho_/I_pho_) increased). The wider the width of the deformed PDMS microstructure was as the mechanical pressure increased, the stronger was the field intensity at the waveguide core-graphene-PDMS interface. Thereby, the output optical power decreased gradually as the vertical force increased. Under a pressure larger than 40 kPa, the optical interaction strength of the guided light beam in the core with graphene did not increase further, owing to the limited mode field diameter of the guided mode.

According to our theoretical prediction, the change in the output light intensity of the TE and TM modes was measured differently. The relative optical power change (*Δ*I_pho_/I_pho_) of the TE mode (open blue triangle) was greater than that of the TM mode (open black square), which means that the optical response of the TE mode to the mechanical stimulation was more sensitive than that of the TM mode. The TE mode light was more absorbed by the single layer graphene owing to the polarization-dependent nature of the graphene’s optical absorption coefficient. The averaged highest *Δ*I_pho_/I_pho_ values for the TM and TE modes were 6.4% and 5.0%, respectively. If the polarization selection was not considered, the sensitivity of the optical response of the device to the pressure would increase by the contribution of the entire polarization light beam. The averaged highest *Δ*I_pho_/I_pho_ value was 14%. Based on the experimental results, we concluded that an optical power attenuation was possible, even when the refractive index of the superstrate was lower than that of the waveguide core.

**Figure 4:**
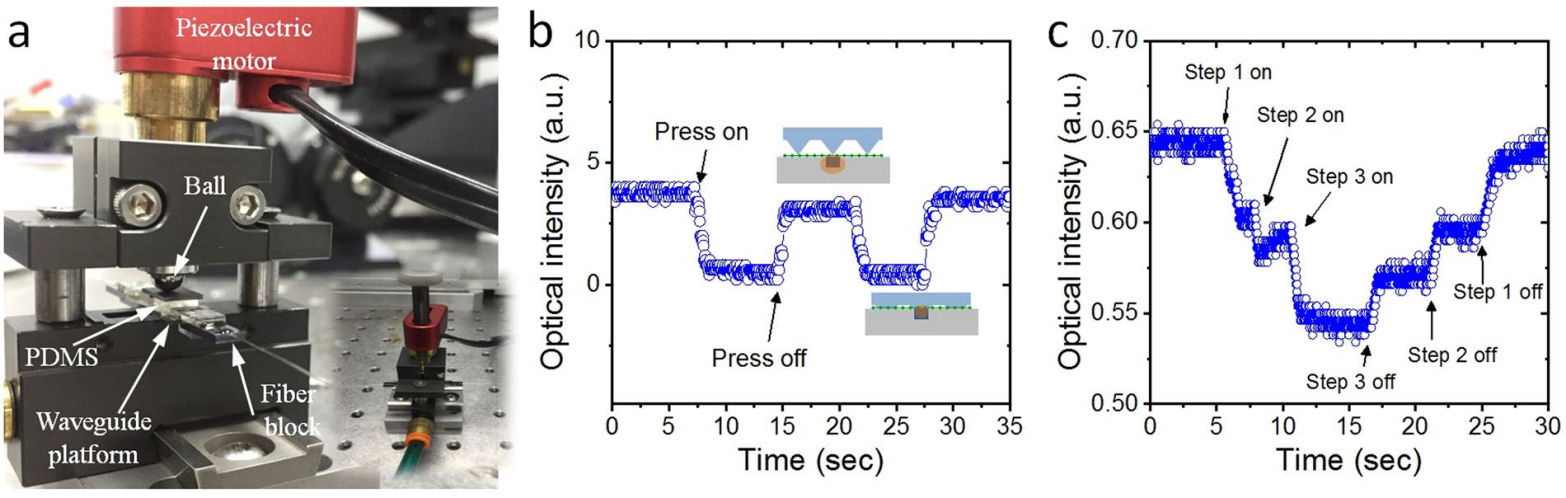
(**a**) Close-up view of measurement setup to measure the dynamic response of the graphene-based optical tactile sensor. The right bottom inset displays the whole system. A mechanical vertical force was applied by a piezoelectric motor. (**b**) Temporal behavior of the mechanical-to-optical transducer according to the dynamic mechanical force. (**c**) The variation in light intensity of the optical device as a function of the stepped movement of the rotator bar.

To measure the dynamic response of the micro-opto-mechanical device to the various changes of the mechanical force, we induced a periodic pressing and release of the PDMS superstrate with an unspecified time interval. Figure 4a shows a close-up view of the measurement setup. The inset displays the whole measurement system. The apparatus was built specifically for the task. A mechanical vertical force was applied by a piezoelectric motor (Newport), whose stepping rotary correspondent to the linear upward and downward movements of the bar that exerted a certain pressure on the PDMS superstrate. A ball was attached at the end of the motor bar for a smooth rotation-to-displacement conversion, and a silicon piece was placed on the PDMS superstrate for uniform pressure.

Figure 4b shows the real-time optical output power during the pressing/releasing cycles. The insets show the cross-sectional view of the device with and without a vertical pressure applied to the low-index microstructured PDMS. As we expected, with the aid of spring-like compressibility and restitution, the periodic variations of the optical output intensity were measured according to the dynamic vertical mechanical force. The optical responses corresponded to the mechanical pressing and release of the superstrate in a timely manner. The fabricated photonic device could serve as an optical modulator. The increasing and decreasing time for the dynamic response is expressed by *W*(*t*) = *W*_0_ + *D*{1 − exp[−(*t* − *t*_0_)/*τ*]} and *W*(*t*) = *W*_0_ + *D*exp[−(*t* − *t*_0_)/*τ*], respectively, where *W*_0_ is the optical power in the ground state, *D* is the scaling constant, *t*_0_ is the gate voltage on/off time, and *τ* is the time constant. We obtained the time constants of 0.35 s and 0.31 s for the increase and decrease, respectively. The decreasing and increasing time of the optical output related to the operation speed of the piezoelectric actuator that we used. The piezoelectric motor could be moved up and down with a maximum speed of 1.2 mm/min, which corresponded to 2 kHz. Therefore, the operation speed of the modulator was several kHz. If we applied faster compression and release to the microstructured PDMS superstrate, a high speed optical power modulator could be configured. Figure 4c shows the variation in light intensity of the optical device as a function of the stepped movement of the rotator bar. The stepwise change of the position of the bar corresponds to discontinuous mechanical pressing and releasing, and exactly correlated to the change of the light output intensity. In addition, the reaction speed coincides with the temporal change of the mechanical pressing and releasing. These experimental results confirm that the proposed optical device can be used as an optomechanical tactile sensor.

## Conclusion

To overcome the shortcomings of the directional coupler-based optical tactile sensor, the refractive index of the upper substrate must be equal to or larger than that of the waveguide core, an optical tactile sensor using a low refractive index PDMS superstrate was proposed and experimentally investigated. By pressing an elastomeric superstrate placed on a graphene-integrated optical waveguide, the prism-like microstructure on the superstrate deformed laterally. This resulted in a wider superstrate interface contacting the graphene film. Thereby, the field amplitude and strength of the guided mode at the lossy graphene-elastomer superstrate interface increased and the intensity of the output optical power was reduced, even when the refractive index of the superstrate was lower than that of waveguide core. The experimentally measured optical responses to pressure were in good agreement with the theoretical predictions. Owing to the polarization dependent light absorption properties of graphene, the reaction of the TE-mode light was more sensitive to pressure than the reaction of the TM-mode light. The optical response to the dynamic pressing and release was a fast and timely matching behavior. In this study, we were able to select a variety of optical materials for the realization of a directional coupler-based optical tactile sensor, without considering its refractive index.

## Methods

### Fabrication of a graphene-integrated polymer waveguide platform

A graphene-integrated polymer waveguide platform was fabricated based on a commercially available ultraviolet (UV) curable resin (ChemOptics). A 30 μm-thick cladding layer and a core layer with a thickness of 7 μm were sequentially spin coated and cured by UV light. Then, the core microstructures with a width of 7 um were defined by using photolithography and an O_2_ plasma dry etching technology. A thin cladding layer was spin coated on the square-defined core structures of the upper-cladding. Then, a monolayer graphene film that was synthesized by the chemical vapor deposition (CVD) method was transferred to the waveguide platform by the lifting transfer method using a poly(methyl methacrylate) (PMMA) supporting layer.

### Fabrication of a microstructured PDMS superstrate

The microstructured PDMS superstrate was separately fabricated using a standard 〈100〉 n-type Si mold. The Si_3_N_4_ layer was deposited onto both sides of the Si wafer by using plasma enhanced chemical vapor deposition (PECVD). Then, the straight lines were patterned by photolithography and the opened Si_3_N_4_ lines were dry etched. After the patterned Si wafer was immerged into 30 wt.% KOH solution, we obtained a smooth Si mold. PDMS, which is commercially available as Sylgard 184, was used. After the sequential fabrication procedures, mixing the silicon elastomer base and curing agent, pouring the PDMS mixture over the Si mold, curing in an oven at 100 °C for two hours, and demolding the cured PDMS from the Si mold, we obtained a microstructured PDMS superstrate. The height of the prism-like structure was approximately 100 μm. The fabricated PDMS superstrate was placed on the graphene-integrated waveguide platform in a flip-chip manner.

### Measurement

To measure the fabricated optical tactile sensor, the unpolarized light from a polarization scrambler (Fiberpro PS3000 series), which received light from a fiber-coupled laser diode (Thorlabs), was launched to the input port of the waveguide sample. Then the output light was collected by a polarization-maintained-fiber. A TE- or TM-mode light was selected by a fiber coupled polarizer (OZ optics) and its optical power was measured by an optical power meter (Wandel & Goltermann). The output power of the unpolarized light was measured without the fiber-pigtailed polarizer, and then that of the TE- or TM-mode was measured separately. A detailed description of the optical measurement set-up can be found in our previous work^18,21^.
